# Guidance on Home-Based Care for People with Severe ME/CFS: A Transdisciplinary Expert Statement

**DOI:** 10.1007/s10354-026-01182-3

**Published:** 2026-07-29

**Authors:** Joachim Hermisson, Claudia Schreiner, Stefanie Weichselbaumer, Marlene Werner, Verena Hackl, Jacob Roth, Sandra Leiss, Anna Christina Maukner, Silvia Wojczewski, Astrid Hainzl, Sabine Hermisson, Kevin Thonhofer, Sabine Pleschberger, Kathryn Hoffmann

**Affiliations:** 1 Austrian Society for ME/CFS, Vienna, Austria; 2 Endowed Professorship in Nursing Science, Center for Public Health, Medical University of Vienna, Vienna, Austria; 3 Department of Primary Care Medicine, Center for Public Health, Medical University of Vienna, Vienna, Austria; 4 Faculty of Mathematics and Max Perutz Labs, University of Vienna, Vienna, Austria; 5 #MillionsMissing Germany, Bedburg-Hau, Germany

**Keywords:** ME/CFS, Schwere Myalgische Enzephalomyelitis/Chronisches Fatigue-Syndrom, Bettlägerigkeit, Pflege, Häusliche Versorgung, ME/CFS, Severe Myalgic encephalomyelitis/chronic fatigue syndrome, Bedridden, Nursing, Home-based care

## Abstract

**Background:**

Many patients with Myalgic Encephalomyelitis/Chronic Fatigue Syndrome (ME/CFS) have significant care needs. However, post-exertional malaise—the defining feature of ME/CFS—means that even minor physical, orthostatic, cognitive, or sensory stressors can trigger a disproportionate worsening of symptoms. This results in specific requirements and significant challenges in home care. Care is still provided predominantly by family caregivers, who frequently lack adequate assistance and support. At the same time, there are significant gaps in knowledge, care infrastructure, and professional guidance for nurses and other healthcare professionals, as well as physicians involved in providing care.

**Objective:**

The objective of this guide is to structure care measures in a way that prevents overexertion and promotes stability.

**Methods:**

The guide is based on a compilation of practice-oriented measures that have proven effective from the perspective of patients and family caregivers. These were professionally categorized and further developed by experts in nursing science, physical therapy, general medicine and public health.

**Results:**

The guide describes how to adapt key dimensions of care—from nutrition and personal hygiene to communication and managing emotional stress—to disease-specific exertion thresholds. Additionally, it outlines requirements for the caregiving relationship and the planning of home visits and discusses the application of palliative care principles.

## Abstract (de)

**Hintergrund:**

Viele Betroffene von Myalgischer Enzephalomyelitis/Chronischem Fatigue-Syndrom (ME/CFS) sind hochgradig pflegebedürftig. Gleichzeitig bedingt die Post-Exertionelle Malaise (PEM), das Kernmerkmal von ME/CFS, dass bereits geringe körperliche, orthostatische, kognitive oder sensorische Belastungen eine disproportionale Zustandsverschlechterung auslösen können. Dadurch ergeben sich spezifische Anforderungen und erhebliche Herausforderungen in der häuslichen Versorgung. Die pflegerische Versorgung wird bislang überwiegend von Angehörigen getragen, die dabei nur unzureichend Hilfe und Unterstützung finden. Gleichzeitig bestehen erhebliche Defizite in Wissen, Versorgungsstrukturen und professioneller Orientierung für die an der Betreuung beteiligten Pflege- und Gesundheitsberufe sowie Ärzt:innen.

**Ziel:**

Ziel dieses Leitfadens ist es, pflegerische Maßnahmen so auszurichten, dass Überlastungen vermieden und Stabilität unterstützt wird.

**Methoden:**

Der Leitfaden basiert auf einer Zusammenstellung praxisorientierter Maßnahmen, die sich aus Sicht von Betroffenen und pflegenden Angehörigen bewährt haben. Diese wurden durch Expert:innen aus Pflegewissenschaft, Physiotherapie, Allgemeinmedizin und Public Health fachlich eingeordnet und inhaltlich weiterentwickelt.

**Ergebnisse:**

Der Leitfaden beschreibt Anpassungen zentraler Pflegedimensionen – von Ernährung und Körperpflege bis hin zu Kommunikation und Umgang mit emotionalen Belastungen – an krankheitsspezifische Belastungsgrenzen. Ergänzend werden Anforderungen an die Pflegebeziehung und die Planung von Hausbesuchen dargestellt sowie Möglichkeiten palliativer Pflegeprinzipien diskutiert.

## 1. Introduction

The aim of nursing care is to enable patients to live a dignified life that is responsive to their individual needs. This typically involves aspects such as communication, social participation, activity, mobility, and physical contact. However, for patients with myalgic encephalomyelitis/chronic fatigue syndrome (ME/CFS), these principles are applicable only within the limits of their highly variable and fluctuating tolerance to exertion, as dictated by the disease. For severely ill patients, in particular, this tolerance is extremely low. Care interventions that benefit patients in other healthcare settings can significantly worsen the condition of patients with ME/CFS and are therefore not applicable. The central clinical characteristic is post-exertional malaise (PEM), which plays a pivotal role in all care-related decisions for ME/CFS [1–4].

This results in a nursing and medical approach that differs fundamentally from established, activation-oriented care models. It requires disease-specific knowledge as well as an awareness of each patient’s individual limits. However, this approach has largely been absent from formal nursing and medical training programs thus far and must therefore be learned specifically. At the same time, the burden on caregivers is considerable. It stems from the extreme demands of caring for seriously ill patients, who often require 24-hour availability, as well as from the severity, duration, and uncertain prognosis of the illness. Additionally, there is often a lack of understanding of the illness within the patient’s social circle, as well as insufficient support from health and social welfare systems. Under these conditions, not only the patients but also the family caregivers often reach—and exceed—the limits of their resilience.

In this situation, clearly structured routines and a personalized care plan are vital tools for providing reassurance to both patients and caregivers and for promoting a sound understanding of the disease. Despite significant individual differences, many caregiving challenges are similar, such as those related to nutrition, oral care, and personal hygiene. Based on practical experience, specific measures and adaptable approaches have been developed for these aspects of care.

These practice-based approaches have largely emerged outside of established care models. Against this backdrop, the experience-based knowledge of patients and their family caregivers becomes particularly important. It forms the basis for adopting *best practices* in care situations that have not yet been adequately addressed.

This care guide draws on this empirical knowledge. It compiles experiences gained over many years and applies them to both informal and formal care.

*This guide is not exhaustive, legally binding, or universally applicable. Rather than a legally binding set of guidelines, it is intended as a professional resource providing guidance on content for care practice. The authors and participating institutions are not liable for any direct or indirect damage, including health impairments or economic losses, arising from the implementation or omission of measures based on this recommendation.*

## 2. Background and development of the guide

The guide was developed by the Austrian Society for ME/CFS in collaboration with the Department of Primary Care Medicine and the Endowed Professorship of Nursing Science at the Center for Public Health of the Medical University of Vienna. The goal was to compile evidence- and experience-based knowledge from professional nursing and make it accessible to caregivers of people with severe ME/CFS.

There remains a significant lack of scientific literature on ME/CFS internationally, particularly regarding the care of severely ill individuals. Patient organizations report significant gaps in healthcare provision as well as a lack of knowledge within the medical community. Patients experience severe limitations in their daily lives and a high symptom burden. Many require care and, due to a lack of recognition and structural integration in the healthcare system, rely almost exclusively on family caregivers. These caregivers are largely left to their own devices, with little guidance or support.

### 2.1 Project group

The guide was developed by a transdisciplinary project group. The group included representatives from the Austrian Society for ME/CFS; individuals living with the condition; family caregivers; and experts in nursing, nursing science, physical therapy, primary care medicine, social medicine, and public health.

### 2.2 Population, users, and objectives

#### Population

The recommendations in this guide apply to adult patients (≥ 18 years) with severe ME/CFS.

The guide is generally applicable in various care settings. However, it has a particular focus on home care, since most people with severe ME/CFS receive care exclusively at home. It focuses on care interventions in the areas of symptom management, basic care, and measures for managing illness-related limitations (pacing).

#### Users

This guide is intended for anyone formally or informally involved in the care and support of people with ME/CFS. In most cases, day-to-day care is provided at home by family members or close friends, so they are a key target group.

At the same time, the guide is applicable to nurses and other healthcare professionals. It can foster a better understanding of the specific needs and challenges faced by people with ME/CFS and their families, help adapt care measures accordingly, and improve care conditions.

#### Purpose of the guide

This guide provides key guidance and recommendations for care of people with severe ME/CFS. It offers basic information on the clinical picture and provides practical recommendations on essential aspects of daily care, such as nutrition, personal hygiene, mobility, communication, and symptom management.

The guide aims to integrate existing scientific knowledge, personal experience of those affected, and practical insights from healthcare professionals to make this information accessible for clinical practice.

The recommendations in this guide are intended as a resource for care decisions. They should always be applied within the context of each patient’s individual situation.

### 2.3 Methodology

This guide is a collection of proven practices from the perspectives of patients, family caregivers, and healthcare professionals. It was developed through a participatory, transdisciplinary process [5]. The goal was to create a resource for the care of people with severe ME/CFS and to generate applicable knowledge through project partner collaboration. This guide does not replace individual responsibility in specific cases. The application of the proposed measures is solely the responsibility of the caregiver.

The role of the scientific partners primarily consisted of providing methodological guidance, structuring the content, and synthesizing the existing literature. The content of the recommendations was shaped largely by patients and family caregivers.

While the guide differs from formally evidence-based clinical guidelines, its planning and organization are based in part on established frameworks (e.g., [6]). Accordingly, it is a structured compilation of practice-relevant recommendations from the perspectives of patients, caregivers, and healthcare professionals. Where possible, these recommendations are supported by existing scientific literature. Alternatively, they were consensually agreed upon within the project group.

A baseline document compiled by the Austrian Society for ME/CFS was used as a starting point. This document was then refined structurally and in terms of content in collaboration with nursing science experts. Since many of the recommendations relate to basic care, the guide’s structure is based on established models and classification systems in nursing science that describe activities of daily living.

Additionally, existing scientific literature on ME/CFS and care was reviewed and incorporated into developing the recommendations (see Table 1 for the most relevant resources).

**Table 1:** Myalgic encephalomyelitis/chronic fatigue syndrome (ME/CFS) and care resources

NICE Guideline on ME/CFS (2021) [2]	British guideline on diagnosis and management; supplemented by practical materials for social care professionals and for comprehensive medical care
Bateman Horne Center Clinical Care Guide (2025) [4]	Comprehensive guide to diagnosis and medical care, with chapters on severely and very severely affected individuals as well as nursing aspects
D‑A-CH Consensus on the Diagnosis and Treatment of ME/CFS (2024) [3]	Authoritative German-language expert consensus on diagnosis and medical care
Textbook of Palliative Nursing (2026) [54]	Addresses the practical aspects of palliative care including symptom management, family support, and psychosocial aspects of palliation

The guide was developed in several sequential steps that exhibit characteristic features of transdisciplinary knowledge generation [7]:

1. Constitutive meeting of the participating organizations, clarification of roles and timeline
2. Definition of the target audience, users, and objectives of the guide
3. Formation of a project group; several working meetings with discussions and negotiation processes regarding structure, content, and literature
4. Iterative revision of the guide and coordination of the recommendations
5. Consensus-based finalization of the content within the project group

#### Timeline and future updates

The guide was developed from November 2025 to March 2026. An update will be released as soon as new, relevant scientific findings regarding the care of people with ME/CFS are available.

## 3. Clinical basics

This section outlines the clinical basics of ME/CFS that are central to care. It covers disease-defining mechanisms, such as post-exertional malaise (PEM), and the principle of pacing. It also covers common symptoms relevant to care, systemic dysfunctions, and comorbidities. The section aims to explain the clinical mechanisms and concepts that are crucial for activity management, symptom monitoring, risk assessment, and the prioritization of care interventions.

### 3.1 Clinical fundamentals of ME/CFS relevant to care provision

Myalgic encephalomyelitis/chronic fatigue syndrome (ME/CFS; ICD-10 code G93.3) is a severe, chronic, multisystem disease. Its defining characteristic is post-exertional malaise (PEM). Various studies estimate its prevalence at 0.3–1% of the population; a significant increase is expected following the COVID-19 pandemic [1, 3]. About one quarter of those affected are homebound or bedbound [1]. Some of these patients depend on care.

ME/CFS predominantly occurs following an infection and is classified as a post-acute infection syndrome (PAIS). Diagnosis is based on internationally recognized clinical criteria, such as the Canadian Consensus Criteria (CCC) [2, 8]. While numerous biomedical abnormalities have been described, no single diagnostic biomarker is currently available for routine clinical use [2, 3].

ME/CFS affects the central and autonomic nervous systems, the immune system, the vascular and endothelial systems, and cellular energy availability and regulation [1, 9]. A characteristic feature is a pathological activity–recovery dysfunction, which leads to a pronounced and often delayed worsening of the condition following exertion (PEM; see Section 3.2).

These disease-related mechanisms cause significant restrictions in physical, cognitive, and autonomic functioning in ME/CFS patients. The spectrum ranges from marked impairments in daily life to a complete dependence on care. Severe ME/CFS causes among the most serious functional impairments in medicine [4, 10, 11].

The literature often distinguishes between four levels of ME/CFS severity: mild, moderate, severe, and very severe (e.g., [2]). However, in a caregiving context, the most important factor is not the formal severity category, but rather the individual combination of symptoms and intolerance to physical exertion and environmental stimuli. These factors often fluctuate significantly over the course of the illness, varying between good and bad days and depending on the time of day. For this reason, and to improve readability, this guide does not distinguish between severe and very severe cases. The focus is on all patients requiring care.

### 3.2 Post-exertional malaise (PEM)

Post-exertional malaise is the central and distinctive hallmark of ME/CFS [1, 8]. It describes a pathological activity–recovery dysfunction in which physical, cognitive, orthostatic, or sensory exertion leads to a disproportionate and often delayed deterioration of the patient’s condition. A pronounced manifestation of this deterioration is referred to as a *crash*, which is not only marked by the intensity of the reaction, but also by an impaired recovery: the body is unable to recover from exertion within a physiologically appropriate timeframe. The deterioration may occur immediately, but typically, the significant effect only becomes apparent after hours or days. A crash can last from several days to weeks, and in severe cases, it may lead to an irreversible decline in functional capacity [2, 4, 8].

Post-exertional malaise is a manifestation of the underlying multisystemic illness and distinguishes ME/CFS from primary deconditioning, nonspecific exhaustion, and stress reactions of other origins [1, 2]. It is central to diagnosis, severity assessment, and all care-related decisions.

A crash triggered by PEM can significantly worsen existing symptoms and/or cause new ones. The tolerance threshold is not static but varies from day to day and is largely determined by the level of prior exertion throughout the day. In addition to a pronounced deterioration in condition (crashes), patients also report less persistent deteriorations (flare-ups). These generally do not reach the severity of a crash but can be precursors and likewise require a systematic reduction in exertion (see [2]).

If overexertion is detected early and the total exertion is consistently reduced, the condition may stabilize and return to its previous functional level. However, full recovery cannot be reliably predicted. Conversely, repeated overexertion can lead to sustained deterioration and a permanent lowering of the tolerance threshold, thereby increasing the vulnerability to further crashes [1, 2, 4].

#### Implications for care

A central goal of care for ME/CFS patients is therefore to consistently avoid crashes. The following should be noted:

- *Overexertion is not immediately visible. *ME/CFS patients may temporarily exceed their individual exertion limits when asked or under situational pressure. Since crashes often occur with a time delay, they are not always correctly linked to the triggering stressor.
- *In severe cases, even minimal stimuli can be overwhelming. *In severe ME/CFS, exercise tolerance is sometimes so low that even basic care interventions (e.g., repositioning, feeding, quiet communication, dim lighting) can trigger a crash [2, 4]. In such cases, repeated and overlapping episodes of deterioration may occur (sometimes referred to as “rolling PEM”; [4, 12]). In this scenario, there is no complete stabilization between individual episodes of exertion, so that the functional capacity remains consistently low and unstable.
- *Deteriorations in condition are multisystemic. *Symptom deterioration usually affects the individual’s primary symptoms (e.g., pain, cognitive impairment, autonomic dysregulation), but it can also trigger new symptoms (e.g., tinnitus that occurs only during a crash). Deterioration cannot be reduced to “exhaustion” and often does not correlate directly with the triggering stressor (e.g., muscle pain following cognitive overload).
- *Stabilization before care optimization. *If the patient’s condition continues to deteriorate or shows no signs of stabilization, the response should not be to escalate measures (“more support”). Interventions (e.g., additional infusions) can exacerbate the situation. Instead, the overall burden should be consistently reduced. This may mean shortening or postponing care procedures, limiting them to an absolute minimum, or temporarily suspending them entirely.

### 3.3 Pacing as a central principle of care

Pacing encompasses all measures and strategies for managing activities, exertion, and stimuli to prevent overexertion and PEM-induced crashes.

Pacing is not a curative therapy and does not automatically improve health status [2, 3]. However, it is the central prerequisite for stabilizing the condition and creating favorable conditions for possible future improvement.

For patients with mild to moderate illness, pacing is primarily a form of self-management within one’s tolerance range. This requires a supportive environment and suitable social and structural conditions, but it is primarily the responsibility of the affected individuals.

For patients with severe illness, pacing increasingly becomes the responsibility of the care team. All care interventions (including those described in this guide) must be evaluated to determine whether they will reduce or unintentionally increase the overall burden in a given case. The following applies:

- *The goal is not activation, but stabilization*. Rather than encouraging patients to do as much as possible on their own, the priority is to minimize exertion. If adequate care resources are available, patients should be encouraged to delegate as many tasks as possible.
- *Stresses accumulate and must be weighed against one another. *Physical, cognitive, emotional, orthostatic, and sensory exertion from activities or care interventions add up and can reinforce one another. Every intervention consumes a portion of limited resources. Therefore, care procedures must always be planned within the context of the total daily exertion. For instance, if a doctor’s visit is scheduled for a given day, it may be necessary to spend the remainder of the day in strict rest to avoid a crash.
- *Rest periods are an essential part of care. *After each care intervention, sufficiently long rest periods are required—ideally in a low-stimulus environment while lying down and without additional demands. For very seriously affected patients, it may be necessary to spread even minor care tasks throughout the day and to schedule longer breaks between steps.
- *Leeway for activities is reserved for quality of life. *Any potential leeway beyond necessary care tasks should not be used up unplanned. These limited reserves are often crucial for quality of life and autonomy, such as a brief contact, a personal ritual, or a small moment of normality.

#### Example of inappropriate care

A caregiver misinterprets a brief period of stabilization as an improvement and increases communication while serving a meal. The patient responds and engages in conversation because it still feels possible at that moment. Hours later, however, she develops severe headaches as well as pronounced sensitivity to light and sound. In the following days, she becomes so weak that even important stabilizing routines, such as her cat’s evening visit, are no longer possible. This example illustrates that even well-intentioned interactions can create a significant additional burden.

### 3.4 Symptoms and comorbidities relevant to care

ME/CFS is characterized by a range of symptoms and comorbidities that significantly influence care provision. They can affect exercise tolerance, sensory processing, nutritional status, circulatory stability, and the response to medications and other external stimuli.

The following overview focuses on core symptoms and comorbidities that are particularly relevant to the care of people with severe ME/CFS, as described in international guidelines and clinical reviews [2–4]. For each individual case, the specific combination of symptoms and their severity are decisive.

#### 3.4.1 Sensory intolerance

Intolerance to light, noise, touch, odors, and/or chemicals is a common symptom of ME/CFS, often becoming extremely pronounced in severe cases. This hypersensitivity can affect multiple sensory modes simultaneously and fluctuate significantly over time. Even minor stimuli may be perceived as painful, overwhelming, or autonomically destabilizing; they may trigger a PEM-induced worsening of symptoms and potentially lead to a crash. Hypersensitivity to stimuli is an expression of the impaired neuroimmunological and autonomic regulation in ME/CFS and must not be mistaken for “hypersensitivity in the psychological sense.”

In severe cases, patients are often unable to protect themselves from stimuli. Even everyday environmental stimuli, such as quiet conversations, diffused daylight, kitchen odors, vibrations, and brief physical contact can be a significant burden.

Many severely affected individuals also exhibit sensitivity to medications. Even low doses can trigger strong or paradoxical reactions. Therefore, medications should be introduced at the lowest possible dose (“start low, go slow”). New medications or other substances should be introduced individually and at intervals to clearly identify the cause of any reactions.

Care interventions should be performed in a way that minimizes stimulation as much as possible. Reducing stimulation is not just for comfort; it is a key prerequisite for stabilization. The implications are wide ranging and affect all aspects of care, including mobility, personal hygiene, sleep, and medical care. The section “4.6 Safety, Protection, and Infection Control” addresses practical recommendations and helpful tips.

#### 3.4.2 Dysautonomia

Autonomic dysfunction is common in ME/CFS and is a significant clinical concern, especially in severe cases. It can affect various organ systems and manifest as orthostatic, gastrointestinal, urogenital, or sudomotor dysfunction [1, 2, 11, 13, 14].

Clinically, orthostatic forms of dysautonomia predominate. Manifestations of orthostatic intolerance (OI), defined as the inability to tolerate an upright posture (sitting, standing, elevating the head) without significant worsening of symptoms, are particularly common. A common manifestation is postural orthostatic tachycardia syndrome (PoTS), in which standing up leads to an abnormal increase in heart rate [2, 3, 13].

In ME/CFS patients with comorbid OI, an upright body position can lead to dizziness, nausea, palpitations, cognitive impairment, weakness, or even a crash due to orthostatic stress. In very severe cases, even slightly elevating the head or sitting for a short time is not tolerated. This can result in substantial functional limitations that can significantly impact care provision:

- Change the patient’s position slowly and gradually and allow for a sufficient recovery period after orthostatic stress.
- Avoid positioning the patient in an upright position unless absolutely necessary. Assess the need for head elevation on a case-by-case basis, and do not use it routinely.
- Adjust care measures to times of better circulatory stability (e.g., in the afternoon instead of in the morning). Prepare transfers carefully, and, if necessary, divide them into several stages.
- Monitor vital signs (heart rate, blood pressure) during orthostatic stress and take fluid and electrolyte status into account.
- Do not misinterpret symptoms as “anxiety” or “deconditioning.”

##### Risk of falls and fall prevention

Since OI can cause sudden weakness, dizziness, and circulatory instability, there is a significant risk of falls. However, a distinction must be made:

- Patients with very severe disease are usually permanently bedbound. In this case, the focus is less on traditional fall prevention and more on safe positioning and avoiding positioning-related incidents.
- For severely ill patients who are still able to move about or go to the bathroom, there is a significant risk of falling. However, it must be considered that in ME/CFS, preventing crashes is the primary goal. Measures to prevent falls must not lead to additional strain.
- For combined fall *and* crash prevention, ensure that standing up or sitting down is done slowly and gradually, guided by symptoms. Allow for an immediate return to a horizontal position if symptoms of dizziness appear.

#### 3.4.3 Gastrointestinal dysfunction and mast cell activation

Gastrointestinal dysfunction is common in ME/CFS and can significantly destabilize the care situation. Symptoms may include delayed gastric emptying (gastroparesis), early satiety, nausea, bloating, abdominal pain, and severe food intolerances.

Additionally, some affected patients exhibit signs of mast cell activation or mast cell activation syndrome (MCAS) [3, 15]. These conditions are characterized by inappropriate release of mast cell mediators, including histamine, and present with multisystemic, fluctuating symptoms. Along with gastrointestinal symptoms, patients often experience flushing; cardiovascular symptoms; skin symptoms; and increased sensitivity to foods, medications, odors, and chemical substances. The severity of a reaction can vary greatly [16, 17]. These gastrointestinal dysfunctions and sensitivities can significantly impair food intake and lead to weight loss, malnutrition, or dehydration.

Recommendations for care provision:

- Identify and systematically document individual triggers (medications, odors, temperature, stress) and food intolerances.
- Spread out food intake and schedule sufficient rest periods. A regimen of 4–5 smaller meals is generally recommended [18] but may not be suitable for all patients.
- Ensure adequate fluid and electrolyte intake.
- Reduce exposure to odors and irritants during meals.
- Have a clearly defined emergency plan for severe reactions and other known complications.

#### 3.4.4 Pain

Pain is one of the core diagnostic criteria for ME/CFS [8] and represents a significant burden in severe cases. The physical and autonomic stress caused by severe pain can trigger crashes, which in turn may further intensify pain symptoms. This vicious cycle may lead to persistent post-exertional deterioration, as recovery is repeatedly interrupted by new exertion-induced worsening (“rolling PEM”), requiring high-priority attention.

Pain can be musculoskeletal, neuropathic, migraine-like, or exertion-induced, and it often fluctuates. Some ME/CFS patients exhibit small-fiber neuropathy (SFN), i.e., damage to the small nerve fibers responsible for pain, temperature, and autonomic functions [19]. Clinically, this can result in neuropathic pain (burning, stabbing, electric shock-like), pronounced sensitivity to touch (allodynia), temperature dysregulation, sweating, circulatory instability, and paresthesia [20].

Recommendations for care provision:

- Take pain symptoms seriously and treat them thoroughly, especially if there is a risk of self-perpetuating deterioration. Do not misinterpret them as hypersensitivity.
- In cases of sensitivity to touch, avoid pressure and friction as much as possible. Prepare the patient for touch with a brief, possibly nonverbal cue to prevent startle responses.
- Pain treatment should be supervised by a physician and will often primarily involve medication (e.g., [3, 21]). However, attention should be paid to individual intolerances and adverse effects.
- Some complementary approaches such as pain-relieving positioning (if tolerated) can be helpful. Others, such as distraction through other stimuli, are typically not suitable.

#### 3.4.5 Joint hypermobility

In some people with ME/CFS, generalized joint hypermobility occurs in association with a hypermobile Ehlers–Danlos syndrome (hEDS) or hypermobility spectrum disorder (HSD) [3]. Note that mechanical instability in hypermobile ME/CFS patients not only causes (chronic) pain but can also promote autonomic dysregulation and thus trigger or exacerbate PEM-associated symptom worsening [2, 3, 22–24]. Therefore, joint protection and stabilization should be considered as part of multimodal and care interventions [23].

Hypermobility affects not only large joints but can also involve smaller structures (jaw, ribs, feet). Even minor stress or unfavorable positioning can trigger pain, subluxations, or crashes. In some patients with pronounced connective tissue weakness, instability of the cervical spine is described, including craniocervical instability (CCI). This may be accompanied by neck pain, headaches, neurological symptoms, dizziness, or autonomic dysfunction [4].

Recommendations for care provision:

- Position all joints, especially the cervical spine, in alignment with their natural axes to provide stability. Avoid forced flexion, extension, or rotation.
- Avoid hyperextension or pulling movements (e.g., of the head when repositioning).
- Perform movements slowly, in a controlled manner, and with a small range of motion. Perform passive mobilization with great caution.
- Ensure additional stabilization during transfers.
- Use supportive aids (pillows, rolls, splints) as needed and take individually prescribed orthoses into account.
- Be mindful of skin fragility (exercise caution with adhesive bandages and electrodes).

Mechanical instability and the resulting pain and PEM-associated symptom worsening should be avoided whenever possible. Passive mobilization or manual techniques involving hypermobile joints, particularly the cervical spine, should only be performed by appropriately qualified professionals (e.g., experienced physical therapists) and only when clearly indicated.

## 4. Areas of care in ME/CFS

The challenges posed by patients with severe symptoms are diverse and affect multiple levels of care:

- Severe physical weakness may be so advanced that basic activities of daily living can no longer be performed independently. These include, among other things, going to the bathroom, holding a cup, chewing, and turning over in bed [11].
- Extreme sensitivity to stimuli (see 3.4.1) means that interventions are only possible under highly restricted conditions, including care in almost complete darkness, with minimal physical contact and maximum protection [11, 17, 25–27].
- Severe forms of OI (see 3.4.2) significantly limit many interventions [11].
- Severe weakness and intolerance to cognitive stimuli can limit verbal communication or prevent it entirely [11, 17, 25]. In some cases, even the presence of another person in the room is intolerable [26].
- Severe courses of gastrointestinal dysfunction and food intolerances (see 3.4.3) can become the central focus of care, may become life threatening, and/or require enteral tube feeding [11, 17].
- In addition, a wide range of complications arise from comorbidities as well as secondary health issues due to being bedbound and the severity of the illness, such as immobility, susceptibility to infection, constipation, osteoporosis, and depression [11].

The following sections provide an overview of typical challenges and possible care interventions across the main areas of care, ranging from nutrition and personal hygiene to managing emotional distress. Since no valid studies on the care of people with ME/CFS are currently available, the recommendations presented are based on the empirical knowledge of caregivers and patients. They thus reflect the current state of care practice.

*It should be noted that post-exertional malaise (PEM) and existing comorbidities significantly influence all areas of care. All measures must be adapted to the patient’s current capacity for exertion and existing stimulus intolerances and thus aligned with the central principle of pacing (see 2.3).*

### 4.1 Eating and drinking

Ensuring adequate energy and fluid intake is often a challenging task for caregivers in cases of severe ME/CFS. On the one hand, being underweight is a common problem. On the other hand, eating can itself be physically exhausting and trigger crashes. Gastrointestinal problems and food intolerances can further complicate nutrition. In such situations, caregivers must carefully balance necessary care with reducing exertion. In very severe cases or crisis situations, this may require weighing between equally threatening consequences. Ensuring a structured and reliable intake of food and fluids is therefore a high priority. Ideally, supportive measures are initiated proactively before they become urgently necessary. Once adequate nutrition and hydration are reasonably secured, this represents an important step toward stabilizing the overall situation.

#### Key challenges

Difficulties arise due to several factors that often occur simultaneously:

1. *Exhaustion and muscle weakness. *Eating is often very strenuous. Due to severe exhaustion, many patients cannot lift their arms, sit upright, or hold their heads up. Even basic functions such as chewing and swallowing may be impaired or lost entirely [28].
2. *Orthostatic intolerance (OI; possibly PoTS). *In extreme cases, patients can no longer tolerate even minimal changes in position or slight sitting up in bed [29] and must consume all food and beverages while lying flat.
3. *Food intolerances and allergies *(often associated with mast cell activation or MCAS). These often require individualized dietary adjustments [15].
4. *Gastrointestinal complaints*. Motility disorders (with symptoms such as difficulty swallowing, nausea, constipation) up to gastroparesis can significantly impair food intake [28].
5. *Distress caused by the presence of others. *If this is a significant source of stress, providing food or beverages can be significantly more difficult or impossible [26, 30].

#### Care interventions

The appropriate interventions depend on the nature and severity of the patient’s functional limitations. The key consideration is always which routine causes the least strain while providing an adequate level of nutrition. Some patients can still eat suitably prepared meals on their own. In cases of severe illness or during crashes, however, food often needs to be spoon-fed or administered via a feeding tube.

- Fixed times and routines provide structure, offer security, and reduce the need for communication. However, they must be flexibly adjusted during crashes.
- Ideally, patients should be able to sit up slightly or elevate their upper body while eating and drinking because this facilitates swallowing and reduces the risk of choking. If this is not tolerated due to OI, food intake must occur while lying down.
- If chewing is possible, finely chopped foods can be placed within reach. Solid, stable foods are easier to control while lying down than liquids or mushy textures. Under suitable conditions, this may enable independent eating.
- If chewing becomes too strenuous, the diet must be adjusted. Some patients can still eat small, soft pieces of food, such as bread without the crust, but many rely on pureed foods or soups. Pureed foods can also aid digestion and be individually tailored to address food intolerances.
- If physical exertion must be reduced further and meals shortened so that only a few sips are possible, care should be taken to ensure a high-calorie diet. Commercially available liquid foods can be helpful in this regard. In cases of food intolerance, the composition and tolerability must be carefully evaluated.
- In cases of severe symptoms due to allergies, MCAS, or food intolerances, a gradual reintroduction of foods is recommended, starting with a basic diet that is well tolerated and as low in irritants as possible. If available, professional guidance from dietitians or allergists is advisable.
- Adequate fluid intake is important. If possible, an easily accessible water bottle or sippy cup should be available. Water bottles should only be partially filled, as full bottles place an additional burden due to their weight. Lukewarm water is often better tolerated and conserves energy. Rehydration fluids can be helpful in cases of OI.

#### Aids and practical tips

- If individuals can eat independently, or if the presence of others is more stressful than the physical effort, simple aids such as lightweight plastic tableware, cups with straws, and non-slip trays can help minimize the energy required for eating.
- Sippy cups assist with feeding or independent eating and drinking and reduce the risk of aspiration (choking). In cases of severe swallowing difficulties, thickening drinks can be helpful. Special thickening agents are commercially available.
- In cases of histamine intolerance, the “SIGHI list” can help identify suitable foods [31].
- In cases of allergies and intolerances, also pay attention to fillers in medications. Some pharmacies offer hypoallergenic formulations.
- Rehydration solutions can also be prepared at home (e.g., WHO solution).

#### Nutrition support

In cases of very severe food intolerances or significant problems with chewing and swallowing, enteral tube feeding may become necessary [26, 28, 30]. A medical evaluation for tube feeding should be conducted early on if there is a risk of malnutrition or persistent inadequate food intake, rather than waiting until critical weight loss has already occurred.

- Under no circumstances should tube feeding be withheld or delayed in an attempt to motivate individuals to eat independently [2, 28, 32].
- A nasogastric tube can be inserted in a home setting by trained professionals, providing significant relief, as patients must no longer actively worry about food intake. However, some patients do not tolerate a nasogastric tube due to sensory sensitivities in the face and throat area. In such cases, alternative forms of nutrition should be considered early on.
- Other tubes (PEG, PEG‑J, etc.) must be inserted in a hospital and entail additional care requirements, such as wound care or the correct medication administration via the tube. Nevertheless, this may be the better option for long-term tube feeding or patients who are intolerant to nasogastric tubes. Training of the primary caregivers by qualified professionals is strongly recommended.
- Enteral tube food must be selected on an individual basis, introduced gradually, and its tolerability must be closely monitored.
- Parenteral (intravenous) nutrition must be considered if adequate enteral nutrition is not possible due to severe gastrointestinal dysfunction. Indication and administration must be carried out exclusively under medical supervision.

### 4.2 Toileting tasks

Solutions for toileting must be tailored to the individual’s functional level and current tolerance limits. For many severely affected patients, using the bathroom is the most significant and unavoidable physical exertion of the day. Accordingly, it is a key focus in the care plan, along with nutrition and fluid intake.

#### Key challenges

1. *Digestive problems* may result from autonomic dysregulation, prolonged bed rest, or medication side effects and can lead to both hard and very soft stools.
2. *Prolonged sitting *in cases of severe orthostatic intolerance can be very stressful or intolerable.
3. *Going to the bathroom*—several times a day—is often too strenuous or entirely impossible for severely affected individuals. Light and noise stimuli can add to the burden.
4. The *use of incontinence pants *for bowel movements in bed can create significant psychological barriers for non-incontinent patients.
5. Providing *assistance with toileting *can be very time consuming. Irregular schedules require constant availability on the part of the caregivers.
6. The *loss of independence *in this area is a sensitive issue and is often associated with feelings of fear and shame. For many affected individuals, this is emotionally distressing.

#### Care interventions

As in all areas, the goal is to provide the greatest possible relief, paying particular attention to psychological aspects. A reliable routine can help patients to tolerate a burdensome procedure.

- Regular schedules are beneficial and should be practiced, if possible. However, this should not require additional energy. Careful documentation of the frequency and consistency of bowel movements is recommended. These records help assess when measures should be taken and evaluate them.
- Regular, soft bowel movements (without diarrhea) are an important goal, as they provide relief and prevent discomfort. Adequate fluid intake is essential. Additionally, if tolerated, a high-fiber diet can be helpful, such as by adding psyllium or flaxseed (if sufficient fluid can be consumed). Small amounts of olive oil (1–2 tablespoons) as well as easily digestible fruits and vegetables can also be helpful. Foods that cause bloating (e.g., legumes, cabbage, and onions) should be limited if they aggravate symptoms [33].
- If this is not sufficient, stool softeners (e.g., macrogol) may be used. Further measures, such as enemas or stronger laxatives, should only be considered if this does not produce a sufficient effect. Consider additional measures after 2–3 days without a bowel movement.
- Less suitable are complementary interventions that are difficult to tolerate due to sensory or chemical stimuli, such as warm, moist wraps/abdominal compresses, essential oils, or abdominal massages, as well as foods that require chewing (e.g., dried figs).
- Mobile patients should be supported as soon as going to the bathroom becomes strenuous, e.g., by providing a place to rest on the way to the bathroom or by using aids such as a lightweight wheelchair or walker. Dimmed lighting reduces sensory stimulation. A footstool can facilitate bowel movements; moist toilet paper or a bidet can aid in cleansing.
- If the trip to the bathroom becomes too taxing, a commode chair or a camping toilet in the room is often the better solution, with help during transfers if needed. A bedpan (and urine bottle, especially for men) allows for toileting while lying down. Since using these items requires some practice, they should be introduced early on, not only when there is no other option.
- The use of incontinence pants may become necessary in severe cases even when there is no incontinence, but even a bedpan is too taxing. For many patients, this presents a significant psychological and practical barrier (i.e., they are unable to use them even if they want to), especially when such aids must be used for the first time during an acute episode of overexertion. A gentle proactive approach, clear routines, and the preservation of dignity and autonomy are particularly important.
- A permanent urinary catheter may be helpful in certain cases, such as when a caregiver’s presence is not tolerated or when there is bladder dysfunction due to autonomic dysfunction [14]. However, since insertion, wear, and cleaning can be problematic for patients sensitive to stimulation, and because catheters carry an increased risk of infection, careful assessment is necessary. Training by qualified nursing staff is required [34].
- The procedure for bedside cleaning should follow a set “choreography” to which all involved must strictly adhere (when to place the pad, turn the patient over, etc.).

#### Aids and practical tips

- When using a bedpan or in cases of occasional urine leakage, it is important to protect the mattress from moisture. One proven solution is to place a waterproof pad directly on the mattress core and a washable mattress cover or topper on top of that to absorb moisture.
- Disposable panty liners with an absorbent function can be helpful during menstruation or for mild incontinence. Some women use special urination aids to urinate in a bottle while sitting or lying down.
- Odor issues can be reduced with special odor-neutralizing products, if tolerated. Fragrances, room sprays, and perfumed products are often not well tolerated and should be avoided.
- A camping toilet can help maintain independence if the trip to the restroom is too strenuous. Camping toilets typically only need to be emptied every 1–2 days. Chemical additives can extend the intervals, but they are not always well tolerated.

### 4.3 Personal hygiene and dressing

In professional care settings, “visible or complete cleanliness” is often implicitly associated with quality care. However, this understanding must be questioned in the context of ME/CFS. Complete personal hygiene should not be the standard for “good care” if it exceeds a person’s energy limits.

A certain degree of cleanliness and personal hygiene is medically necessary; contributes significantly to subjective wellbeing; and plays an important role in maintaining the dignity, identity, and self-worth of those affected. In severe ME/CFS cases, however, the priority is preventing a worsening of the condition, even if this requires reducing the usual care routines.

Therefore, it is essential to work with patients to determine which care procedures they can tolerate. When doing so, consider that patients may sometimes overestimate or overstate their capacity due to shame, habit, or politeness. Abandoning familiar care routines (e.g., regular hair washing) can also cause emotional distress. Caregivers should actively support the adjustment process and try to normalize it as much as possible. They should watch for signs of overexertion (changes in breathing, paleness, slowed reaction) and respond accordingly. In practice, they are more often “advocates for pacing” than “guarantors of cleanliness.” The goal is not to maintain external standards, but to protect against deterioration while preserving dignity.

#### Key challenges

1. *Showering and hair washing *pose significant physical strain for many individuals (orthostatic stress, temperature changes, sensory stimuli).
2. *Marked sensitivity to touch *can make personal care considerably more difficult or even impossible at times.
3. *Regular nail care and shaving *present particular challenges.
4. *Having others provide care* and *reduced personal hygiene *can be perceived as a loss of autonomy, intimacy, and dignity, causing significant psychological distress.

#### Care interventions

In severe ME/CFS, personal hygiene is not focused on completeness, but on medical necessity and individual exercise tolerance. Procedures are based on general care standards that aim to ensure skin and mucosal integrity as well as preventing infection [35]. At the same time, ME/CFS-specific guidelines emphasize strict adherence to exertion limits to prevent post-exertional symptom exacerbation [1, 2].

An adapted minimal personal hygiene regimen pursues three goals:

1. Infection prevention (intimate hygiene, oral hygiene, cleaning after contamination).
2. Maintaining skin integrity (including preventing pressure ulcers and inflammation).
3. Preserving dignity while minimizing physical exertion as much as possible.

Any additional measures must be considered on a case-by-case basis and carried out only if they are tolerated.

- Prepare each step thoroughly to minimize the time spent in the room. Prioritize care tasks and perform them in a calm and structured manner within a limited timeframe.
- Partial body washing in bed is the gentlest option for severely affected patients. In such cases, “minimal washing” (face, hands, genital area) or cursory washing with a damp cloth may be sufficient. Strenuous activities, such as lifting the arms or turning in bed, should only be performed within a safe tolerance range.
- Moderately affected patients may still be able to shower with a seating aid and assistance. When transferring to the bathroom, particular attention must be paid to the risk of falls, especially in cases of weakness and OI (see 3.4.2).
- Regular skin inspections should be performed, particularly on vulnerable skin areas: sacral region, heels, elbows, shoulder blades, back of the head, and skin folds [35]. Ideally, this should be done as part of routine care procedures to avoid additional strain.
- Despite patients being bedbound, clinical experience shows that pressure ulcers occur less frequently in ME/CFS than in many other conditions that cause immobility. However, they must still be monitored. Persistent redness that does not fade under light pressure indicates tissue damage and requires immediate pressure relief [35]. If any damage is suspected, professional advice should be sought.
- Persistent moisture due to sweating, incontinence, or extended contact with damp fabrics can lead to skin softening (maceration). Macerated skin is susceptible to infections because it provides favorable breeding ground for bacteria and fungi. After cleansing, gently pat the skin dry with a soft cloth (do not rub) and pay particular attention to skin folds [35].
- *Skin protection*: Apply creams only if desired or medically indicated. In cases of excessive moisture (e.g., due to heavy sweating), apply a zinc oxide barrier protection to skin folds. For very dry skin, use pH-neutral, perfume-free products [35]. As little as possible, as much as necessary.
- Wash hair in bed (e.g., using a shower cap or inflatable tub) only if expressly desired and tolerated safely.
- Trim hair or beard at longer intervals (weeks or months). Use stable periods and spread the process over several short sessions if necessary. Keeping hair short makes care significantly easier.
- Check finger- and toenails regularly for infections, fungi, and risk of injury, and trim them at least at longer intervals (in stages, if necessary, trim only a few nails per day). For very hard or ingrown nails, consult a professional pedicurist.

#### Aids and practical tips

- Hair-cutting tools (razors, electric shavers, or [beard] scissors) should be adapted to individual needs, depending on whether the duration, noise, or tactile stimuli are perceived as particularly stressful.
- *Combing and scalp care*: Short hair usually does not require combing. Soft massage brushes can help with an itchy scalp.
- When washing hair, using a support or cushion for the head (e.g., a bath pillow) can reduce strain.

#### 4.3.1 Oral care

Dental and oral problems are common and clinically significant secondary complications in severe ME/CFS, especially when self-care is limited and visits to the dentist are impossible. Inadequate oral care can lead to oral infections, tooth decay, and pain. Inflammatory processes in the oral cavity are also associated with systemic effects [36, 37]. However, although essential, oral care also represents a significant burden in severe ME/CFS.

#### Key challenges

1. *Brushing teeth is often quite taxing*, especially when done thoroughly. Light, noise, and vibrations can add to the burden.
2. *Taking over a patient’s dental hygiene requires practice*. It may take time to develop a good routine.
3. *More extensive dental treatments *(e.g., fillings) are usually unavailable for patients with severe conditions.

#### Care interventions

The scope and intensity of oral care are guided by medical necessity and the individual’s exertion tolerance.

- For oral care in bed, raise the head of the bed slightly and collect spit in a kidney basin or other suitable container.
- Thorough brushing once a day (ideally in the evening) is more beneficial than multiple brief brushings. Fluoride toothpaste (if tolerated) is recommended for dental health [38]. Antibacterial mouthwashes can be used in addition, provided they are well tolerated. Interdental brushes or dental floss should only be used if tolerated.
- A fixed routine (e.g., inner/outer, upper/lower) can help minimize communication during brushing. Whenever possible, inspect the oral cavity for redness, plaque, pressure points, and signs of infection.
- If desired, a division of tasks can be agreed upon and incorporated into the routine. For example, patients may be able to better identify remaining plaque in low light, so it sometimes makes sense for them to finish cleaning certain areas themselves.
- If possible, arrange for dental home visits for visual inspections. If caries or inflammation is suspected, act early, as later treatments can be significantly more stressful. In the early stages, intensive fluoridation can be effective. Suitable products should be discussed with dental professionals.

#### Aids and practical tips

- If tolerated, electric toothbrushes can make cleaning easier. Ultrasonic toothbrushes are quieter, but they vibrate more strongly. Individual tolerance is the deciding factor.
- For dry mouth, try frequent small sips of water or sugar-free lozenges. If necessary, moisturizing gels or saliva substitutes can provide additional relief.
- Caregivers without experience in assisted oral care should familiarize themselves with the technique and procedure in advance. If possible, they should practice on volunteers.

#### 4.3.2 Dressing and bedding

Changing pajamas and bedding can be taxing for people with ME/CFS. Establishing set routines and avoiding too frequent changes can make things easier.

#### Key challenges

1. *Putting on and taking off pajamas *requires lifting and moving the limbs as well as turning in bed.
2. *Changing the bed sheet with the patient in bed *requires a practiced routine and repeated turning. Blankets and pillows can be changed more easily.
3. Changing linens is particularly difficult if the patient is *sensitive to touch.*
4. *Impaired thermoregulation *(feeling too hot or too cold) may require frequent adjustments of the blankets.

#### Care interventions

- If necessary, open tops at the back (like a winged shirt) and secure them at the neck with laces or clasps. This allows for a change without turning over and (almost) without lifting the arms. Change tops daily or at longer intervals, depending on tolerance.
- Choose loose-fitting bottoms and ideally change them during toilet visits. One-piece garments and nightgowns are impractical. Underwear is usually unnecessary. If necessary (e.g., during menstruation), slips with clasps can be helpful.
- Change bed sheets only when necessary and tolerated. The routine should be adapted to the circumstances. The typical procedure is to carefully turn the patient onto one side, partially remove the old sheet and prepare the fresh one, then carefully roll the patient over the folded sheets in the middle, and complete the change. If short periods out of bed are possible (e.g., using a commode chair), these should be utilized. With a good routine in place, two people can perform a sheet change very quickly (in about 30 s).
- Keep freshly made blankets and pillows ready, prepared outside the room.
- For clothing and bedding, ensure that the materials are well tolerated, particularly regarding sensory sensitivity and impaired thermoregulation. Soft bed socks may be used if needed and tolerated.

### 4.4 Rest and sleep

Sleep dysfunction is a key symptom of ME/CFS [1, 2]. It is characterized primarily by unrefreshing sleep, but also by severe difficulty falling and staying asleep. Another common feature is a shift in the day–night rhythm, with sleep often lasting into the late morning and wake phases extending well past midnight. For severely affected patients, the situation is exacerbated by the fact that, in addition to sleep, “resting while awake” in a dark room is often the only viable alternative. Thus, the bed is not only a place for sleep. It is also the living space almost all the time. Despite the limited restorative function, getting enough sleep remains an important goal in symptom management. Even unrefreshing sleep is preferable to persistent sleep deprivation and can help stabilize the condition.

#### Key challenges

1. A strongly *shifted day–night rhythm *can complicate care planning, especially when regular care tasks (feeding, toileting) are necessary late in the evening or after midnight.
2. *Sleep dysfunction *in ME/CFS is not primarily behavioral in nature but is rather associated with an interplay of autonomic dysregulation, neuroinflammatory processes, mast cell activation, and pain symptoms [4]. Accordingly, general sleep hygiene measures are usually insufficient.
3. The *bed *is the environment in which severely affected individuals spend not only the night but often 95–100% of the day. It must meet their diverse needs and enable *comfortable care *for both the patient and caregiver.

#### Care interventions

Traditional sleep hygiene recommendations (e.g., “go to bed only to sleep,” “do not rest during the day”) are often contraindicated or not feasible in severe ME/CFS. Instead, the following goals take priority:

- Adapt care planning to individual sleep phases whenever possible. Accept a stable individual rhythm and do not actively attempt to normalize the shifted day–night rhythm.
- A familiar bedtime routine (e.g., a fixed sequence of evening care or a short personal ritual) provides a sense of closure and security and can facilitate the transition from wakeful rest to sleep.
- Ensure a comfortable and stable room temperature.
- Many patients require sleep medication. Prescription and selection must be made by a physician. Caregivers assist in monitoring effectiveness and tolerability. Information on substances used in ME/CFS treatment can be found in specialized treatment guidelines (e.g., [3, 21]).
- In cases of being completely bedbound, a fully adjustable nursing bed enables independent positioning changes and allows for gentler care for patients and caregivers alike. A temporary solution is an electrically adjustable slatted frame with a remote control.

#### Aids and practical tips

- Essential items (e.g., water bottle or sippy cup, call bell) should be placed within easy reach. A wider bed helps to increase the storage space.
- If the mattress is uncomfortable, a washable mattress topper is a good first measure.
- A clearly visible clock and, if desired, a calendar can help keep track of time.

### 4.5 Mobility, positioning, and transferring

Patients with ME/CFS who require care are almost always housebound and often permanently bedbound. Prolonged immobility and bed rest increase the risk of contractures, pes equinus, and pressure ulcers, all of which require special attention. Mobility in the traditional sense, by contrast, is usually not a goal, but represents a significant stressor and health risk. Depending on the situation and severity, however, affected individuals sometimes perform regular transfers (e.g., to the bathroom or toilet or between rooms) independently. Even for bedbound patients, situations may arise that make a change of location unavoidable, such as necessary medical procedures, renovation work, or hazardous conditions (e.g., mold or fire). In such cases, transportation should be safe and involve minimal exertion, even if a crash cannot always be avoided.

#### Key challenges

1. Regular *repositioning*, which is necessary in cases of a high risk of pressure ulcers and to prevent contractures and *pes equinus*, can cause exertion and thus poses a challenge.
2. Many patients are unable to move independently, even with the aid of a wheelchair, due to *extreme weakness *and/or intolerance to physical exertion.
3. Patients with *severe OI *(e.g., in cases of PoTS) can only be transported while lying down.
4. Simply *leaving the protected environment *(bed, bedroom) can trigger deterioration, even leading to a crash.
5. *Stairs *pose a significant obstacle, even with assistance.

#### Care interventions

- Activities beyond the daily routine that carry a significant risk of triggering a crash must be carefully planned and well prepared to ensure that everything goes smoothly.
- For short-distance transport and within the home, an ultra-light travel or transit wheelchair is suitable, provided there are no stairs to navigate. For short transports in a lying position, transfer sheets are recommended. If possible, these should be carried by four people to minimize jolts. Ideally, this procedure should be practiced beforehand with a healthy person to ensure that everything goes smoothly in an emergency.
- For longer trips or in cases of OI, a wheelchair should have a reclining function (reclining backrest and adjustable leg rests).
- Transportation by car should, whenever possible, be carried out in a lying position using a medical transport service. Special needs, such as blackout curtains, noise protection, and a smooth driving style should be discussed in detail beforehand.
- If tolerated and prescribed by a doctor, the preventive administration of sedatives can dampen sensory processing and thus reduce the risk of a crash.
- *Positioning interventions*: Measures, including those for contracture and pes equinus prevention, should only be performed if they are tolerated and will not trigger a crash due to touch, movement, or a change in position. If regular repositioning is necessary for pressure ulcer prevention (ideally assessed by nursing staff), caregivers must learn gentle turning techniques to relieve pressure and avoid shear forces and incorporate them into their routine. All caregivers should use the same maneuvers to minimize additional strain. In cases of generalized joint hypermobility, individually customized positioning cushions or rolls can help stabilize joints (see 3.4.5).
- To prevent falls and reduce strain, use devices such as wheelchairs, rollators, and walking aids. Keep the environment free of tripping hazards. If necessary, additional seating options can be used to provide relief.

#### Aids and practical tips

- When purchasing a wheelchair, consider weight, maneuverability, and a ride that is as vibration free as possible. Telescoping rails can assist with short staircases or loading heavy wheelchairs (e.g., electric). Transfer boards or sliding mats can be useful for transfers between the bed and wheelchair.
- Stair-climbing aids that do not require modifications to the stairwell are available either as devices with an integrated seat or as systems that transport wheelchairs.
- Many assistive devices, such as wheelchairs and stair climbers, can also be rented, which is ideal for trying out or for occasional use.
- Emergency sheets or disposable transfer sheets are cost effective and should always be kept within reach for permanently bedbound patients for rapid evacuation, e.g., in the event of a fire.
- Alternating-pressure mattresses for pressure ulcer prevention are often not tolerated due to noise and pressure changes.

### 4.6 Safety, protection, and infection control

In severe ME/CFS, safety encompasses more than traditional fall or infection prevention. Any additional stress or stimulus, whether infectious or sensory, can trigger a PEM-induced worsening of symptoms. In this context, safety therefore means, above all, protecting patients from avoidable exertion while ensuring they receive the care they need.

#### Key challenges

1. *Infections *pose a high risk of long-term health deterioration.
2. *Illnesses among the care team *can significantly jeopardize care provision.
3. Severely affected individuals are usually unable *to adequately protect themselves from external stimuli.*
4. *Sensory sensitivity *(to light, noise, touch, odor, temperature, chemicals) can be extremely pronounced and make caregiving very difficult.
5. *Cleaning, ventilation, and maintenance tasks *can themselves become a burden.

#### Care interventions

##### Infection control and exposure prevention

- In the patient’s room, the care team and visitors must wear well-fitting FFP2 or FFP3 masks. If you are ill, avoid direct contact entirely if possible and arrange for a substitute.
- Maintain infection control measures also outside the patient’s room (wear masks indoors, ensure good indoor air quality, receive recommended vaccinations).
- Consistently disinfect hands before every care measure and before food preparation.
- Ventilate the room as often as possible (considering sensitivity to light and noise). A HEPA air purifier may also be used, if tolerated.
- Make vaccination decisions for affected individuals on a case-by-case basis, considering prior experiences and indirect protection via herd immunity.
- Cleanliness in the patient’s room, changing of bedding, and personal hygiene contribute to infection control but must be adjusted according to the patient’s tolerance.

##### Protection against sensory overload

- Sunglasses or eye masks are often necessary even in low light and help increase tolerance levels. Try out different models and make sure to have enough masks in reserve.
- Noise protection via headphones or earplugs must also be tried out individually. Noise-canceling headphones can be useful but are not always tolerated (e.g., pressure on the head and ears, aggravated tinnitus).
- Care must often be provided with minimal light and, if necessary, in total darkness. This requires practice and—especially here—adherence to a strict routine.
- Reduce everyday noises as much as possible and avoid noisy tasks during a crash. Announce unavoidable noise in advance and specify a reliable timeframe (e.g., “5 min of drilling”). Intense but brief noise is often better tolerated than prolonged exposure. Medication to reduce sensory overload may be considered in consultation with a doctor.
- Do not use perfumes and shield the room from strong odors (e.g., from the kitchen). Depending on tolerance, use only fragrance-free detergents and personal care products. If there is a severe intolerance to chemicals, avoid detergents and cleaning products as much as possible.
- If there is a choice of rooms, the most important criterion is a quiet location (noises and voices from inside and outside the apartment). Good darkening and ventilation to prevent mold are also important. Air conditioning can regulate temperature and humidity. Hard floors are easier to maintain than carpets.
- Cleaning should be done infrequently and as briefly as possible, if necessary, only in short intervals of 1–2 min. If available, a spare room is ideal for occasional deep cleaning or renovations, provided that a transfer (e.g., wheelchair, transfer sheet) is possible.

#### Aids and practical tips

- Custom-fitted ear protection can be very helpful. The fitting (by a specialist at home) takes just a few minutes. However, both the brief procedure and the necessary light must be tolerated.
- *Targeted lighting*: A headlamp with a focusable beam is practical for tasks that only require illumination of a small area, such as trimming toenails.
- Electric blinds and remote-controlled systems for regulating light, temperature, and humidity provide patients with a basic level of control and independence. Early adaptation, while the condition still allows, is advisable. Many of these measures are beneficial already at moderate disease severity.

### 4.7 Communication, social interaction, and presence of people

Communication is an indispensable basic human need. In severe ME/CFS, however, opportunities for communication are severely limited and require individual adaptations.

#### Key challenges

1. *Processing language *requires significant cognitive effort, which is often even more taxing for severely affected individuals than simple sounds.
2. *Written text *also activates the language center (“you can’t not read”) and is therefore often perceived as taxing.
3. The mere *presence of people *in the room can be cognitively taxing, even if they are not speaking. This is especially true for visits outside of the routine, which can trigger strong emotions (even joy can be stressful).
4. Individuals at the very severe end of the spectrum are often *too weak to speak or write*. Furthermore, communication requires concentration, which is often severely limited.
5. *Cell phone use* may cause crashes.
6. At the same time, *complete social isolation *can be emotionally very stressful.

#### Care interventions

First and foremost, it is essential to maintain the most important communication channels. All measures against social isolation should be developed together with the affected person and tailored to their needs.

- For many patients, their cell phone is their last valuable connection to the outside world. For this reason, exceeding the tolerance threshold poses a significant risk. A time limit or complete abstinence may be necessary. The decision rests solely with the patient, at least in the case of adults. Technical limits on usage time can be helpful when agreed upon.
- If the cell phone is used to communicate with the care team, alternatives should be established to enable temporary or permanent abstinence.
- Basic communication should be nonverbal: e.g., a call button to alert a caregiver, gestures, finger signals, or simple sounds for “yes” and “no.” It is important to have a clear “stop” signal, after which all activities are stopped as quickly as possible, and the caregiver leaves the room.
- Establishing additional signs is advisable and should be prioritized at the first signs of language loss, e.g., signs for *thirst (drink), bathroom, good/bad, too cold/warm, too loud/bright, emergency, pain, thank you.* Customized symbols can also be used to express emotions (e.g., sad or happy faces) and to represent important people (including the care team and doctor). When discussing body parts *(where does it hurt?)*, an avatar—such as a doll or stuffed animal with arms and legs—can be helpful. Pictures (e.g., small cards) are often better suited than text, which strains the language center.
- Limit communication from the care team to what is necessary, using a few clear sentences or individual words that have been carefully considered beforehand. Once a routine has been established, a discussion about the care process is often unnecessary. Important messages (e.g., schedule changes) can be conveyed with single words or gestures. Ask yes/no questions. Nonessential communication (e.g., greetings, news from the world) should only take place at appropriate times and upon the patient’s request. Setting times for communication helps patients prepare in advance, reducing stress.
- There should never be more people in the room than necessary. In cases of very low tolerance, individual solutions must be found. As much as possible, patients should be able to eat on their own and wash themselves with wet wipes to avoid additional stress. If this is not possible, sedative medication may be used to provide care in exceptional cases.
- Visits are exhausting but are sometimes important for overcoming social isolation. The decision rests solely with the patient and must be respected. Visitors should be prepared for the possibility that they may not be allowed to enter the room at short notice, even if they have traveled far to do so. Visits may only be possible for a very short time, and they may need to proceed without verbal communication. Visitors should always be announced in advance, though it is often better to do so at the last minute to avoid prolonged tension. Short, ritualized routines (e.g., fixed visit duration, clear roles, minimal speech) can reduce cognitive strain while providing emotional support through reliability.

#### Aids and practical tips

- A wireless bell with multiple receivers that can be placed throughout the house is usually a good solution for calling a caregiver.
- In extreme cases, when even pressing a button is no longer possible, a baby monitor may be an alternative.

### 4.8 Emotional and existential needs

Severe ME/CFS places patients in an extreme psychological situation. Massive physical and cognitive limitations, constant dependence on assistance, repeated flare-ups and crashes, and neglect by healthcare and social services systems lead to significant emotional distress. Feelings of despair, anxiety, panic, hopelessness, and being overwhelmed are common and expected under these conditions. Depressive symptoms may also occur. However, all these symptoms and phenomena are generally the result of ongoing distress, not the root cause of the illness. Psychological reactions must not be misinterpreted as an explanation for ME/CFS or any PEM-related deterioration.

Models such as Fennell’s four-phase model describe coping processes during chronic illnesses [39]. However, for severely affected individuals who do not experience any meaningful improvement in symptoms, the “integration” or “acceptance” phases (Fennell phases 3 and 4) are often unattainable, or only partially attainable.

#### Key challenges

1. Unlike many physical stresses, psychological stresses are often nearly impossible to avoid. Therefore, they are among the most common crash triggers for severely affected individuals.
2. Lack of knowledge about ME/CFS—and particularly about PEM—among therapists or those in the patient’s support network can lead to misdiagnoses and harmful interventions (e.g., encouraging activity in cases of alleged or actual depression).
3. Toxic positivity (*You must not give up hope! You have to fight!*) increases the pressure on patients and can trigger feelings of guilt. This is often associated with forms of *victim blaming*—the implicit or explicit assumption that patients are responsible for their own situation or are not doing enough. This can also occur in a medical context as so-called “medical gaslighting,” which occurs when symptoms are questioned or trivialized [40, 41]. Conversely, complete acceptance of the situation as the “highest stage of coping” is not a realistic goal for individuals with very severe ME/CFS.
4. Limited communication options make it difficult for patients to express their emotional state in a nuanced way or to ask for help.
5. Phases of extreme or prolonged distress can trigger existential crises; patients may also express wishes to die or end their own lives.

#### Care interventions

- Consider emotional stress as a relevant trigger of crashes. As part of the general stress management strategy (pacing), emotional stress should be reduced as much as possible to prevent emotional overload. The focus is on helping patients cope from day to day, not on processing or resolving emotional issues.
- Shield patients from stress triggers as much as possible. These triggers may include, for example, rejection letters from government agencies, distressing medical information, or contact with people who lack an understanding of the illness. Ideally, patients will explicitly delegate emotionally demanding tasks to their care team or family members.
- Coping techniques can help manage stressful situations. For severely affected patients, these techniques are usually quite simple, such as ritualized daily routines, brief moments of positive attention, or a short personal mantra.
- Calm, deep breathing can help with pacing during times of emotional stress and hyperventilation. Patients should be aware of their breathing and, if possible, be able to control it. Appropriate schemes are often minimal and flexible; rigid breathing therapy techniques are usually too taxing.
- Medications (antidepressants or, in acute situations, sedatives) help some patients. Discuss their use with a physician, ideally one with experience in ME/CFS. They should not be viewed as curative therapy; rather, they should be evaluated based on whether they help to reduce emotional overload and contribute to stabilization.
- Professional psychotherapeutic support can be helpful if
  - the patient wants it,
  - the therapist has experience with ME/CFS, and
  - communication and stress limits allow for it.
  All interventions must be PEM aware and not trigger crashes. Activating or confrontational approaches should be avoided.
- Supervision or case conferences for caregivers can be helpful, especially in persistently stressful situations or when dealing with existential issues.

#### Dealing with/responding to a wish to die

In cases of very severe ME/CFS, extreme stress and crisis situations may arise, raising existential questions and desires. In this context, a wish to die may emerge as a response to overwhelming suffering. Findings from palliative care show that such wishes can vary in intensity and duration, often including ambivalent elements whereby a wish to die and the desire to live can coexist [33]. A wish to die may be situation-specific and intensified by acute stress or may reflect an expression of an ongoing existential crisis. The latter is by far the more common scenario in severe ME/CFS. In these situations, crisis-sensitive, respectful support is important, as is the early involvement of palliative care and supportive specialist services. This guide does not explore legal and ethical issues in depth, but they should be clarified on a case-by-case basis with the relevant specialist agencies.

- Actively inquire whether the person is experiencing thoughts of death, expressing a wish to hasten death, having suicidal thoughts, or has developed a concrete suicide plan. There is no evidence that addressing such thoughts triggers or exacerbates them [33].
- Do not judge, do not trivialize, do not over-dramatize. Maintain a respectful, open, and empathetic attitude. It is not necessary to provide answers or solutions. Simply being present and enduring the situation together is also an effective form of support [33].
- If there are indications of acute suicidal ideation (e.g., increasing urge to act, concrete plans),
  - take them seriously;
  - if possible, do not leave the person alone without support, while also considering the coping limits of family members;
  - involve existing support (family members, medical contacts, crisis services) at an early stage;
  - assess whether distressing physical or sensory factors can be reduced (see [33]).
- Consider distressing physical symptoms as potential triggers. Pain, shortness of breath, restlessness, anxiety, and severe sensory overload can intensify thoughts of death. Assess whether better symptom relief is possible in these cases.
- When a wish to die is expressed repeatedly, it is important to be familiar with relevant end-of-life options. However, the focus should be on responding respectfully to the person’s expressed wishes, continued observation, and offering support through conversation and appropriate sources of help.

### 4.9 Roles in family and partnership

Severe ME/CFS fundamentally alters the roles of individuals within partnerships and families. Patients may be unable to fulfill their roles as partners, parents, or family members, while their primary caregivers take on new responsibilities. These changes can trigger grief, feelings of guilt, and tension, and they should be recognized as part of the caregiving experience. New ways of interacting must be learned. Love and empathy toward patients with severe ME/CFS are demonstrated through reliable, diligent, and persevering care, as well as consistent support in managing the disease through pacing.

#### Key challenges

1. With severe ME/CFS, the usual *rules of loving and respectful coexistence *must be relearned to some extent. Relationships are put to the test and may fall apart if they cannot adapt to the conditions imposed by the illness.
2. Unlike many other chronic illnesses, the *intensity of the limitations *(physical, cognitive, orthostatic, sensory, emotional) leaves little room for maintaining relationships unaffected by the disease (“intrusiveness of ME/CFS” [39]).
3. The *caregiver–patient dependency *relationship is challenging for both parties.
4. *Conflicts *are a natural part of any relationship. However, in cases of severe ME/CFS, conflicts often cannot be resolved through discussion.
5. Professional and personal limitations may result in a *loss of social status *for family caregivers or a lack of understanding (*What’s in it for you?*). This can lead to psychological distress and even secondary traumatization [39], often with repercussions for patients, who feel like a burden.

#### Care interventions

- Trust is the foundation of every relationship. Building this trust requires that patients are believed. This is especially true when it comes to information about their symptoms. Patients must be able to count on their limited communication being taken seriously and conveyed reliably.
- A sense of security and comfort arises from reliability and understanding. Good care routines form the foundation. Beyond that, personal rituals offer an opportunity to foster familiarity and express mutual affection. Rituals can express different roles, such as those of a parent or partner, and they can help keep memories and hopes alive. They should be developed together and must not exceed the patient’s limits. For the most severely affected patients, rituals are often nonverbal and may be limited to individual gestures or touches that are woven into caregiving activities.
- Trusted caregivers also serve as protectors and pacing assistants. In emotionally charged situations or during sudden bursts of activity, patients may exceed their exertion limits without realizing it in time. Experienced caregivers should then point this out and remind them to take breaks. Rules can be agreed on about when to remind patients about pacing.
- Contact with other ME/CFS patients in similar situations can be a very valuable resource. In the most severe cases, however, this is only possible to a very limited extent and requires the active support of the care team.
- If possible, primary caregivers should also communicate their own distress or grievances. When doing so, they should manage their own irritability outside the room whenever possible. Frustration within the care team should be addressed through mutual support, including relief from caregiving duties during periods of acute overload.
- Since caring for ME/CFS patients can be a long-term commitment, family caregivers should also be aware of their own limits. Stabilizing the care team is just as important as medical care for everyone involved, including the patients themselves. Good communication within the team and networking within self-help groups are essential.

## 5. Guidelines for healthcare professionals

This section describes the specific requirements for providing home-based care to people with severe ME/CFS and outlines key care principles, structures, and practical recommendations. Although it is primarily intended for nurses, it also offers other healthcare professionals practical guidance on caring for this highly vulnerable group of patients.

Caring for patients with severe ME/CFS requires a fundamental shift away from established nursing paradigms. Rather than focusing on activity promotion and a resource-oriented approach, the primary goal is the consistent prevention of overexertion. Post-exertional malaise, profound physical weakness, and sensory intolerance define the boundaries of all nursing care. Interventions that are considered beneficial in other clinical contexts may lead to sustained deterioration in people with severe ME/CFS and should therefore not be used.

Instead, care follows a person-centered care approach that, in the context of ME/CFS, includes the principles of harm prevention, stimulus reduction, and minimal intervention. This includes precise planning, a significantly reduced frequency of interaction and communication, and the consistent adaptation of all care interventions to the individual’s highly restricted and fluctuating limits of exertion [4, 27]. For professional caregivers, this restrained approach is often counterintuitive and emotionally taxing [27]. It requires specific expertise, consistent self-control, and a high degree of empathy. An approach based on palliative care principles can provide valuable guidance in this context.

Only under these conditions can a care environment be created that facilitates stabilization and, in some cases, may also allow for functional improvement. However, the course of the disease varies from person to person. Even with optimal care, improvement cannot be guaranteed [4, 11].

Care for severely affected ME/CFS patients—except for emergencies—typically consists of brief interventions spread throughout the day. This places special demands on the planning, communication, and coordination of care. Clear structures, reliable procedures, and continuity of staff are essential prerequisites for a safe care environment in the home setting. Equally important is a trusting care relationship.

### 5.1 Building a trusting care relationship

In cases of severe ME/CFS, a stable and reliable relationship between caregivers, patients, and their families is essential [4, 11, 42]. Due to their high vulnerability to exertion and external stimuli, ME/CFS patients depend heavily on the sensitivity, attentiveness, and adaptability of their caregivers [11, 25, 42, 43]. Only through a stable, trusting relationship can individual exertion limits be recognized, care routines be implemented, and crashes be avoided [11, 25, 43, 44].

The most important prerequisite for such a relationship is conveying empathy, sincerity, and reliability in nursing practice [11, 43]. This includes the serious and nonjudgmental acknowledgment of reported symptoms and the consistent alignment of nursing interventions with individual tolerance limits. It also requires a willingness to deviate from familiar, standardized nursing procedures [4, 11, 25, 43].

Similarly, a sustainable care relationship requires a nursing approach tailored to the severity and complexity of the illness. Central to this approach is a comprehensive understanding of how the disease affects all aspects of life, including the physical, psychological, social, and existential or spiritual dimensions. It also involves being transparent about the limits of one’s knowledge and a commitment to staying informed about the latest ME/CFS research and guidelines [2, 11, 43].

Severe ME/CFS can result in a complete loss of one’s former life and sense of self [11]. Therefore, a particularly sensitive approach is required for patients and their families, allowing space for loss and grief while offering support and reliability. Caregivers play a key role in ensuring that patients feel heard, taken seriously, and supported. This is especially important given their repeated experiences of trivialization and pathologization [25, 43, 45].

#### Family members and primary caregivers as an important resource

A collaborative and trusting partnership with family members and primary caregivers is particularly important for home care of people with severe ME/CFS [4].

Primary caregivers are those who, together with the patients, develop individualized care routines over extended periods of time. A nuanced understanding of day-to-day variations in tolerance, early signs of overexertion, and individually tolerable care interventions does not develop through isolated home visits but through continuous observation, careful trial and error, and repeated adjustments in everyday life. For those with very severe symptoms, even basic care tasks, such as personal hygiene or repositioning, can only be performed in short intervals with longer recovery periods. Such individualized care can rarely be provided within the structured and often rigid schedules of professional care services. The involvement of continuously present caregivers, typically family members or 24-hour care providers, is therefore indispensable [46].

In addition, direct communication with patients during home visits is often limited and, in extreme cases, nearly impossible. In such cases, communication should focus on the most essential issues. Information regarding objective aspects, such as medication or observations about the progression of the illness, can be provided by trusted individuals, usually family caregivers. However, it is important that patients decide on the communication channels and who will represent them. Ideally, this is documented through a formal power of attorney.

At the same time, family members and primary caregivers need guidance, professional feedback, and emotional support. Family members caring for people with severe ME/CFS often assume responsibilities that extend well beyond traditional caregiving duties. They coordinate medical appointments; document medications and symptoms; organize daily life; and represent the patient’s interests within the healthcare, social, and educational systems. They also provide continuous emotional support while witnessing the progression of the illness and its unpredictable, severe symptoms firsthand [4].

Added to this are significant health, social, and financial burdens, particularly in the context of the often-necessary round-the-clock care. Many family members reduce their working hours or quit their jobs entirely, which can lead to long-term financial uncertainty. At the same time, the constant physical and emotional strain increases the risk of health problems and psychosocial overload. Access to welfare and social support services is also often difficult, which can further complicate matters [4, 27, 47].

Against this backdrop, professional care plays a central role. Rather than providing direct nursing care, it involves coordination, organization, and advisory tasks. Experienced nursing staff should guide, evaluate, and—where necessary—adjust and re-instruct the care routines. Equally important is the proactive identification of potential signs of caregiver burnout as well as—where possible—the provision of appropriate respite care, counseling, and support. This protects not only the caregivers but also ensures the stability and continuity of the overall care situation [4, 42].

### 5.2 Home visits

A safe home visit to people with ME/CFS requires careful planning, clear communication beforehand, and a structured on-site approach.

Since patients’ capacities can fluctuate greatly, the timing, scope, and specific implementation of the measures must be reassessed on the day of the home visit and adjusted as necessary. Even with careful preparation, it may be necessary to shorten, postpone, or completely cancel the visit.

#### Planning and preparation

In advance, it should be clarified to what extent the patient wishes and is able to participate in organizational decisions. If possible, feedback should be obtained directly, for example via a brief message or a formal authorization of a representative. In practice, coordination often takes place through family members or primary caregivers who are familiar with the individual’s situation (see Section 3.2).

The following aspects should be assessed in advance:

- Maximum tolerable length of stay in the room,
- light tolerance (darkness, dim light),
- tolerance of communication,
- tolerance to touch,
- tolerance to odors or people in the room.

These factors determine the workflow, staff scheduling, and preparation of materials. Alternative forms of communication, such as written messages or communication via family members, are generally less stressful than direct conversations before or after a care procedure. The time of day for home visits should be chosen based on what is most manageable for the patient.

#### Procedure and conduct in the care room

On the day of the visit, final coordination with the primary caregivers or, with the person’s consent and if feasible, with the person affected should take place before entering the room. Lighting, positioning, placement of supplies, and division of tasks must be clarified. In general, as much preparation as possible should be done outside the room to minimize the time spent inside.

- Within the care room, care should be provided calmly, systematically, and efficiently. Tasks should be completed in a clearly prioritized and logical order to avoid interruptions or corrections.
- A clear emergency signal should be agreed upon in advance that the patient can use to indicate an onset of deterioration (e.g., hand signal, bell, agreed-upon word). If this signal is given, all activities must be stopped as quickly as possible, and everyone should leave the room.
- Invasive procedures (e.g., blood draws, catheter care, or tube management) should be performed exclusively by experienced and skilled nursing staff. Do not assign trainees or those with limited practical experience to ME/CFS patients. Informal caregivers should not be trained directly on the patient but rather using a model or simulation (e.g., for tube care).
- Feedback from previous interventions should be obtained in advance to avoid recurring problems.
- For invasive nursing procedures, premedication may be used as a supportive measure if indicated by a physician.
- Extensive blood draws can be a significant burden for severely affected patients. The following precautions are recommended [12]:
  - Prepare blood draws thoroughly (including spare needles and collection tubes) and perform them during the patient’s “best” time of day, if possible.
  - Due to reduced circulating blood volume (caused by dysautonomia), consider additional fluid replacement (e.g., 1–2 glasses of an electrolyte drink or unsweetened coconut water), if necessary.
  - In cases of severe pain and touch sensitivity, a topical local anesthetic can be applied to the puncture site beforehand.
  - Use a hypoallergenic bandage (or cotton wool secured with Micropore tape) at the puncture site.
- Strict infection prevention and control is mandatory when caring for individuals with ME/CFS. The relevant measures are outlined in Section 4.6.

### 5.3 Care planning and emergency measures

A detailed and well-structured care plan forms the basis for reliable collaboration between the patient, the primary care team, and professional caregivers. A care plan for severe ME/CFS is based on established care routines between patients and their primary caregivers developed over extended periods of time. The aim is for all caregivers (informal and formal) to be able to carry out all routines confidently without having to ask the patient for clarification. Even if patients are still able to communicate verbally, they should not have to expend energy on repeated explanations, which should be reserved for additional, genuinely important communications.

A comprehensive care plan for ME/CFS includes:

- *A medical assessment *including diagnoses; comorbidities; primary symptoms; and all known factors that may trigger, worsen, or alleviate symptoms.
- *Personal information *about the individual, such as interests, hobbies, and previous occupation. This helps convey a realistic picture of the person who was active and participated in social life before the illness. This makes it easier to avoid misunderstandings and misconceptions.
- *Current medication*. Also include previous experiences with medications, including tolerability.
- *In the main section*: clear *details on all care procedures *(time of administration, order, and steps), medication, and communication options. Procedures such as assistance with eating, personal hygiene routines, and specific protocols for pain and other symptoms should be documented in detail.
- *A notes section *allows for recording missing items or changes. The care plan should be reviewed regularly and updated as needed.

#### Emergency measures and interventions

The care plan should have a separate section that includes clear procedures for emergencies and non-routine stressful situations. Examples include home visits by doctors, therapists, or assessors; complex care procedures; and transfers with a high risk of crashing.

- For emergencies, clarify in advance who bears *primary responsibility *and is authorized to make decisions if necessary. These individuals should be reliably available around the clock.
- It should be documented which *physicians familiar with the situation *should be contacted in an emergency (when and how) and whether home visits can be arranged. If complications arise, it is often advisable to *contact them early on *to avoid having to rely on an emergency service at night or on weekends that is unfamiliar with the situation.
- For healthcare professionals without experience in ME/CFS, an official information sheet (such as [48]) can be helpful. Ideally, this sheet would be supplemented and adapted to each case, making important considerations and specific requirements immediately apparent.
- It must also be clearly documented which *medications*, in what dosage, may be administered in emergencies or in preparation for stressful procedures. Benzodiazepines are often used for this purpose. Due to the potential risk of dependence, side effects, and tolerance development, their use should always be discussed with a physician. For transfers, it should also be clarified in advance whether sedation is necessary and how it should be administered.

#### Documentation of the disease’s progression

Since ME/CFS is often associated with significant symptom fluctuations, systematic documentation of the disease’s progression is very important. This also allows for an evaluation of the effectiveness of new medication and of the tolerability of certain care activities (e.g., washing hair).

- Ideally, there should be a *daily record* of the type and severity of symptoms, as well as any crashes. Patients with moderate symptoms may often still be able to manage this themselves by keeping a symptom diary. For patients with severe symptoms, this responsibility is transferred to the care team.
- Depending on individual needs, food and fluid intake, dietary changes, body temperature, and bowel movements can also be documented.
- *Reactions to medications and dietary supplements, *as well as *follow-up monitoring*, should be carefully documented and included in the care plan. To facilitate the attribution of any reactions, it is recommended to start new medications at staggered times.

### 5.4 Elements of palliative care

Palliative care describes an “*approach that improves the quality of life of patients (adults and children) and their families who are facing problems associated with life-threatening illness. It prevents and relieves suffering through the early identification, correct assessment and treatment of pain and other problems, whether physical, psychosocial or spiritual.*” (WHO, [49]).

This definition applies palliative care to conditions with life-threatening or life-shortening courses. While ME/CFS is not primarily characterized by a terminal course, it may lead to a life-threatening situation in severe cases. Furthermore, there are currently no causal or curative therapies available. Given the complex symptom burden and highly challenging care situation for people with severe ME/CFS, the question arises as to what extent attitudes and approaches from palliative care may be appropriate and helpful for this patient group.

Generally, a *palliative care approach *appears particularly well suited to the care of people with severe ME/CFS. This approach is characterized by appreciation for patients’ values and preferences and by a strong *focus on their needs and problems *[50]. Cicely Saunders (1918–2005), the founder of the hospice movement, fundamentally transformed pain management with the total pain concept [51], a holistic perspective based on the idea that “pain is what the patient says, it hurts” [52]. Given the high vulnerability and wide variation in manifestations of severe ME/CFS, such unconditional respect for patients’ individual experiences is of central importance (see also Section 5.1).

Another important aspect of palliative care is the *holistic view *of patients, which also involves the *interaction among different professional groups and individuals*. When working with patients, especially those experiencing existential suffering, a *compassionate yet professional attitude *is more helpful than a distant, primarily hierarchical approach.

Furthermore, palliative care explicitly focuses on the quality of life not only of patients, but also of their families [49]. As discussed in Section 5.1 (“Family members and primary caregivers as an important resource”), family members often bear the main burden of care. At the same time, they are key sources of knowledge in the care process. Therefore, approaches that *systematically identify and address their need for support *are particularly important [53].

Handbooks and textbooks on palliative care provide valuable guidance for managing distressing symptoms and situations (see [54–56]). It should be noted that palliative care concepts were developed primarily in the context of late-stage oncological diseases. Therefore, they are often not directly applicable to ME/CFS but can offer important points of reference in an adapted form. The palliative principle of a “radical focus on the patient’s needs” applies to the *reduction of stimuli and stress*: interventions must be guided by pacing (Section 3.3) and consistently adhere to individual exertion limits. Approaches to *symptom relief*, such as managing pain, nausea, and illness-related anxiety, are relevant in this context and provide guidance but must be carefully assessed at an individual level. However, palliative care interventions cannot always be applied directly, they may require adaptation or may not be appropriate. For instance, interventions that rely on conversation or involve sensory stimulation or touch (e.g., aromatherapy) can trigger adverse reactions in many patients and are therefore contraindicated.

In addition, services from hospice care can play a supportive role in addressing spiritual and existential questions as well as specific challenges in caregiving, both for patients (if possible) and for family members and primary caregivers (see, e.g., International Association for Hospice & Palliative Care, IAHPC, or the European Association for Palliative Care, EAPC [57, 58]).

Palliative care specialists from various professional fields have expertise that can support people with severe ME/CFS (see [59]). A consultation may be helpful, provided that the specialist is knowledgeable about ME/CFS and post-exertional malaise (PEM), and this should be clarified in advance. An overview of resources and worldwide services can be found on the IAHPC and EAPC websites [57].

More in-depth engagement by palliative care professionals with the specific characteristics of ME/CFS could further improve the quality of care. This guide can serve as an initial point of reference for this purpose.

## Data Availability

All data supporting the findings of this work are included in the article.

## Abbreviations

hEDS: Hypermobile Ehlers–Danlos syndrome
HSD: Hypermobility spectrum disorder
ICD-10: International Classification of Diseases, 10th Revision
MCAS: Mast cell activation syndrome
ME/CFS: Myalgic encephalomyelitis/chronic fatigue syndrome
OI: Orthostatic intolerance
PEM: Post-exertional malaise
PoTS: Postural orthostatic tachycardia syndrome
SFN: Small-fiber neuropathy
